# Evaluating the Efficacy of the Marburg Heart Score to Triage Patients Presenting With Chest Pain in an Emergency Department: A Prospective, Multicenter, Observational Study

**DOI:** 10.1155/emmi/6085679

**Published:** 2025-05-20

**Authors:** Loïc Druilhe, Lucie Creusier, Jérémy Pasco, Julie Eloi, Virginie Furet, Eric Roupie, Richard Macrez

**Affiliations:** ^1^Department of General Practice, University of Tours, Tours, France; ^2^Caen University Hospital, Polyvalent Medicine Department, Caen, France; ^3^Clinical Research and Innovation Unit, Centre Hospitalier Public Du Cotentin, Cherbourg, France; ^4^Emergency Department, Robert Bisson Hospital, Lisieux, France; ^5^Emergency Department, Jacques Monod Hospital, Flers, France; ^6^UNICAEN, Emergency Department, Caen-Normandie University Hospital, Normandy University, Avenue de la Côte de Nacre, Caen, France; ^7^UNICAEN, INSERM UMR-S U1237, Physiopathology and Imaging of Neurological Disorders (PhIND), GIP Cyceron, Institute Blood and Brain @ Caen-Normandie (BB@C), Normandy University, Caen, France

**Keywords:** acute coronary syndrome, chest pain, emergency department, Marburg Heart Score, triage

## Abstract

**Objective:** Chest pain is a common complaint in emergency departments. Although most patients are admitted to emergency department intensive care, only 12% have acute coronary syndrome. An accurate, efficient score is needed to improve triage and prevent unnecessary referrals to emergency department intensive care. The Marburg Heart Score, validated to rule out acute coronary syndrome in primary care, is quick to administer and does not require test results. This study aims to assess whether the Marburg Heart Score is effective in a triage setting for patients presenting with chest pain in emergency departments.

**Method:** This prospective, observational, multicenter study was conducted with triage nurses in four hospitals in France between July 15, 2018, and May 31, 2019. The primary endpoint was the negative predictive value of the Marburg Heart Score ≤ 2 for ruling out acute coronary syndrome. Acute coronary syndrome diagnosis was made using medical record data combined with a diagnosis from the physician in charge.

**Results:** A total of 1045 patients were included. For a cutoff score of ≤ 2, the negative predictive value for suspected acute coronary syndrome was 95.6% (95% CI [94.0–97.2]) and the area under the curve was 0.603 (95% CI [0.521–0.685]). There were 28 false negatives, two of which were due to the score being completed incorrectly.

**Conclusion:** This study reveals that the Marburg Heart Score is an efficient tool to direct patients presenting with chest pain and MHS < 2 to a conventional ED bed. This could potentially optimize triage in the emergency department to prevent overloading the emergency department intensive care.

**Summary**

• The Marburg Heart Score (MHS) includes 5 questions: female > 65 or male > 55 years, pain worsening with exercise, pain not reproducible by palpation, patient assuming the pain is cardiac and vascular disease.
• In the primary care setting, it has a negative predictive value (NPV) of approximately 97% for ruling out acute coronary syndrome (ACS).
• Using a cutoff of ≤ 2 for triage in the emergency department (ED), the MHS has an NPV of 95.6% for ruling out ACS.
• This is the first time the score has been used for triage in the ED.
• MHS may help optimize triage of patients with chest pain; however, due to the risk of missing early identification of patients with STEMI, ECGs must be performed within 10 min of arrival.
• Patients with a score ≤ 2 and without ST-segment elevation may be transferred to conventional emergency care.

## 1. Introduction

Chest pain represents 10% of all consultations in European emergency departments (EDs) and 4.7% in the United States [1, 2]. Chest pain may be benign intercostal pain or a sign of a life-threatening condition or acute coronary syndrome (ACS). Among patients who present with chest pain at the ED, ACS is responsible for an estimated 5.1% of cases in the United States and 12% in Norway [2, 3]. Furthermore, a European study showed that among the patients hospitalized for chest pain, only 25% have confirmed ACS [1].

Any patient who presents with chest pain should have an electrocardiogram (ECG) performed within 10 min of their arrival in the ED, according to the 2013 French Society of Emergency Medicine and the 2020 European Society of Cardiology guidelines [4, 5]. However, the setup time and monitoring needed to perform an ECG are often difficult in an ED setting. Also, guidelines recommend that patients with confirmed ACS are admitted directly into an intensive care bed within the ED (ED-ICU) where possible [4]. However, only 25% of patients with ACS have access to this optimal care pathway in France, possibly because a lack of resources makes triage decisions within the ED difficult [6, 7]. Yet, every patient with suspected ACS cannot be systematically directed into very high resource-demanding ED-ICU. This would block critical intensive care services and delay treatment for other critically ill patients.

Therefore, there is a need to provide triage nurses with an efficient tool to identify patients presenting with chest pain who are at a high risk of ACS and are transferred to an ED-ICU bed or at a lower risk of ACS and could be monitored in a conventional ED bed. If a subsequent abnormal ECG or troponin result is found, the patient could then be easily redirected to an ED-ICU bed. The Marburg Heart Score (MHS) is currently recommended to screen for coronary heart disease in primary care, which is excluded at a score ≤ 2 with a negative predictive value (NPV) of 97.3%–97.7% [8–11]. This score could be used to triage patients presenting with chest pain in an ED. However, the MHS has not been used to rule out ACS in patients presenting at an ED with chest pain.

This study aims to assess whether using the MHS to evaluate patients with chest pain accurately rules out ACS in triage to an ED triage setting.

## 2. Methods

### 2.1. Study Design and Participants

This prospective, observational, multicenter study was conducted between July 15, 2018, and May 31, 2019, in four hospitals in Normandy, France: one regional reference hospital and three local hospitals (Local Hospitals 1, 2, and 3). The characteristics of each study center during the inclusion period are summarized in Table 1.

Importantly, the regional tertiary center was the only center with both a cardiologist on call 24 h a day 7 days a week and onsite coronary angiography.

The study included any willing adult presenting with chest pain, who was admitted to one of the four participating EDs during the study period. Any patient younger than 18 or who had trauma-related chest pain or incomplete data was excluded. Also, patients brought to the centers by a Mobile Emergency and Resuscitation Service (SMUR) were excluded as the patients have already been triaged by an emergency physician, thus bypassing the ED admission procedure.

### 2.2. Measurements

Once admitted, the triage nurse calculated the MHS for each patient presenting with chest pain admitted to the ED. The MHS contains five yes or no questions: patient age and sex female ≥ 65 years or male ≥ 55 years, pain worsening with exercise, pain not reproducible by palpation, and the patient assuming the pain is cardiac and known coronary heart disease, cerebrovascular disease, or peripheral vascular disease. Each positive response is awarded 1 point, with 0–2 points being low risk, 3 points being intermediate risk, and 4–5 points being high risk [11].

Four of the study authors diagnosed ACS based on patient medical record data including the clinical examination, ECG, serum troponin, transthoracic echocardiogram, and coronary angiogram, if available, in conjunction with the diagnosis made by the ED physician in charge of the patient. An ECG was considered modified if it showed ST-segment elevation, ST-segment depression, T-wave abnormalities, or a branch block [5]. The fourth universal definition of myocardial infarction and the ESC guidelines were used to establish the diagnosis of ACS [5, 12].

### 2.3. Outcome Measure

The primary endpoint was the NPV of an MHS ≤ 2 for ruling out ACS. The secondary endpoint was to compare the MHS and ACS diagnosis between patients referred to the ED by the emergency call center and those who came via a general practitioner (GP), physician, or self-referral.

### 2.4. Statistical Analysis

The population is described with categorical variables and is presented as total numbers and proportions. Continuous variables were presented with a mean and standard deviation. The comparisons were calculated using the chi-square test. If the sample sizes were less than five, Fisher's exact test was applied. *p* values less than 0.05 were considered statistically significant. For the primary endpoint analysis, the NPV of an MHS ≤ 2 ruling out ACS in a triage setting was calculated for the total study population and for each center separately. We also calculated positive predictive values (PPVs), sensitivity (Se), specificity (Sp), and likelihood ratios with 95% confidence intervals (CIs). To assess the overall discriminative power of the MHS, Se was plotted against Sp to calculate the area under the curve (receiver operating characteristic curve, AUC). These calculations were performed with XLSTAT software and R 4.2 software. For the secondary endpoint analysis, the MHSs and ACS diagnosis were compared according to referral source using Student's *t* test and the chi-square test of independence using IBM SPSS 26 software for the sample description and the secondary endpoint analysis.

Any patient with missing data was excluded from the study. The data were anonymized, in compliance with the RGPD 2017 regulations.

To minimize potential sources of bias and ensure accurate reporting, this observational study was developed and reported according to the STROBE guidelines.

## 3. Results

### 3.1. Population

**Figure 1:**
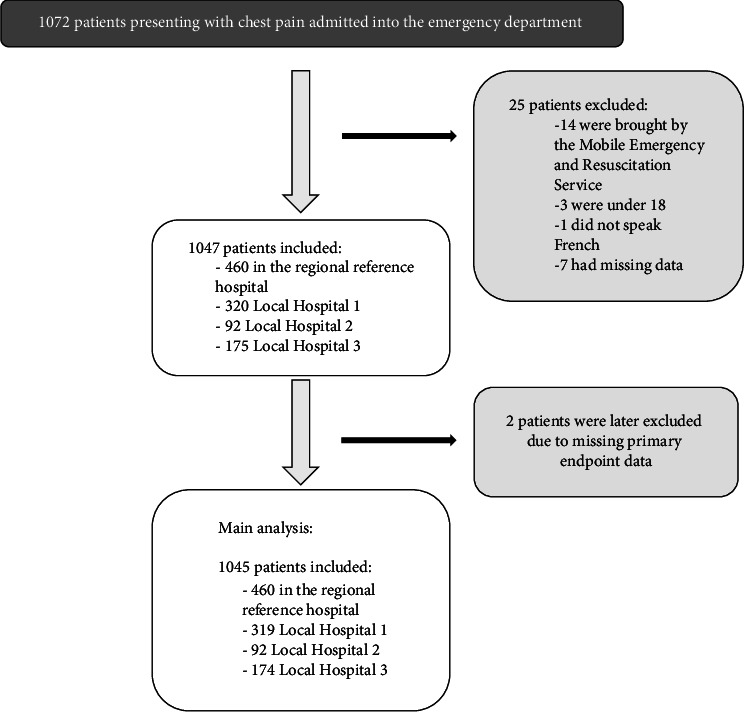
Flowchart of the study.

In total, 1072 patients were enrolled in our study between July 15, 2018, and May 31, 2019. Of which, 14 were brought to the ED by the SMUR, three were under 18 years of age, one did not speak French, and seven had missing data, leaving 1047 patients included. However, primary endpoint data were missing for two patients who were subsequently excluded, leaving 1045 patients for analysis (Figure 1).

The mean patient age was 53 years [52–54] and 576 (55.1%) were men. There was no significant age difference between the centers. Most patients (*n* = 754, 72.2%) were admitted to the ED via a GP, physician, or self-referral, whereas 291 (27.8%) were referred from an emergency call center. Among the 1045 patients included, 24.4% (*n* = 255) had no cardiovascular risk factors, 33% (*n* = 345) were smokers, and 27.4% (*n* = 286) had hypertension (Table 2).

In the regional reference hospital, 20% (*n* = 92) of patients had known ischemic cardiomyopathy which is significantly higher than the overall rate (14.8%, *n* = 155, *p* < 0.001). Furthermore, there was a significantly greater proportion of patients with atrial fibrillation in the regional reference hospital (*n* = 39, 8.5%) compared with the overall population (*n* = 62, 5.9%, *p*=0.022) (Table 1).

In total, 14.4% (*n* = 151) of patients had a modified ECG, 10.5% (*n* = 111) had troponin levels above the center's normal range, 8.9% (*n* = 93) underwent a coronary angiogram, and 9.3% (*n* = 97) had a final diagnosis of ACS.

### 3.2. MHS Performance

At the MHS cutoff value of ≤ 2, the overall AUC was 0.603 (95% CI [0.521–0.685]) and the NPV for ruling out ACS in a triage setting was 95.6% (95% CI [94.0–97.2) (Table 3). The Se, Sp, NPV, PPV, and likelihood ratios are presented in Table 3.

Concerning the secondary endpoint analysis, the MHS was significantly higher for patients referred by the emergency call center compared with those who came to the ED through GP, physician, or self-referral (2.43 + 1.25 vs. 2.09 + 1.19, respectively). In addition, the proportion of patients diagnosed with ACS was higher in patients referred by the emergency call center compared to those who came to the ED via a GP, physician, or self-referral (13.7% [*n* = 40] vs. 7.6% [*n* = 57]) (Table 4).

There were 28 false negatives. Of these, six patients had an ST-segment elevation myocardial infarction, 20 had a non–ST-segment elevation myocardial infarction, and two were due to errors made by the nurse when completing the MHS.

## 4. Discussion

This study shows that the MHS can help optimize the triage of patients presenting with chest pain. Using the cutoff value of < 2, the MHS had an NPV of 95.6% for ruling out suspected ACS in a triage point within the ED. Had this score been applied to the care pathway, 58% of patients in this study would have been directed to a conventional ED bed. Twenty-eight patients who had a false-negative result would have been quickly identified with ECG and troponin levels and redirected to an ED-ICU bed. Indeed, in the 4 centers taking part in this study, all patients consulting for chest pain had an ECG within 10 min of admission, regardless of their referral sector. Interestingly, patients referred to the ED by the emergency call center had a significantly higher mean MHS and a higher rate of ACS compared with patients who were referred from a GP, physician, or self-referral. Furthermore, this score was easy to use, taking only 30 s to complete and only 28 false negatives and two errors occurred among the 1045 patients included.

The NPV of 95.6% for the MHS for triage in an ED is very close to the initial Bösner studies in primary care (NPV of 97.3% and 97.7%) [11]. The ED settings could explain the slight difference. Furthermore, considering that this study was conducted in EDs, it is possible that a higher proportion of the population have had ACS as they either had been referred for suspected ACS or had self-referred due to concern about their chest pain. In total, 9.3% patients (*n* = 97) had a final diagnosis of ACS although only 8.9% (*n* = 93) underwent a coronary angiogram because the benefit-to-risk ratio was unfavorable for some older patients with significant comorbidities. However, the coronary angiogram rate was similar to the 9.8% rate in the SFMU EpiDoulTho study [13].

In contrast to the present study, which found the MHS useful for triage, previous trials of the MHS as a diagnostic aid were inconclusive. A flash-mob study in the Netherlands revealed an NPV of 88% and concluded that the MHS could not safely rule out ACS. However, this study had a low power with a small population of 258 patients [14]. Similarly, a retrospective study also found an NPV of 88% and concluded that the MHS was an unsuitable diagnostic tool in acute primary care [15]. Nevertheless, a 2018 meta-analysis revealed that the MHS was the only score to outperform clinical judgment alone when used in patients with intermittent chest pain in a low-risk setting [16]. Furthermore, the MHS was found to improve the accuracy of GP clinical judgment [10]. These findings are backed up by the 2013 European Society of Cardiology (ESC) guidelines which state that the MHS should be used in combination with other clinical information in primary care [17]. However, the ED is not just for low-risk patients and is different from primary care, so the MHS should be used with caution, as a triage tool, not a diagnostic one, and always with an ECG within 10 min of admission.

The HEART score, used in conjunction with a 1-h high-sensitivity cardiac troponin algorithm, is associated with a reduction in the admission rate, but it requires troponin blood sampling [5], while the MHS is a triage score whose main purpose is not to make a diagnosis but to prevent congestion in the emergency intensive care unit. In a hospital survey including 719 emergency points in 2023, less than 50% of reception conditions at the entrance to EDs were judged to be sufficient, contributing to overcrowding in EDs. The MHS can be a tool for promoting fluidity at the EDs entrance [18].

The MHS is useful to triage ACS to an ED-ICU means it could potentially be used in practice to triage patients scoring < 2 to a conventional ED bed rather than an ED-ICU bed. As this was an observational study, patient management was not modified. Interestingly, when patients were referred to the ED from an emergency call center, they had significantly higher mean MHS and ACS rate than GP or self-referral. This highlights the central role of the emergency call center in optimal patient referral, in which the MHS has recently been shown to reduce the number of unnecessary referrals without compromising triage safety [19]. However, a prospective, interventional study is required to confirm MHS accuracy as a triage tool to evaluate the number of patients transferred to the appropriate department for their diagnosis. In the case of an interventional study, it is important to take into account the risk of false negatives and to have a suitable protocol so as not to delay the management of a NSTEMI patient who has been misdirected at reception.

### 4.1. Strengths and Limitations

It is difficult to generalize the results in the absence of an interventional study. Indeed, if false-negative patients are referred to the conventional ED, they may experience a delay in treatment that can have serious consequences.

This study presented weaknesses and biases typical of an observational study. There was an attrition bias due to the exclusion of two patients with missing data. However, it is unlikely that these exclusions would have changed the outcome, considering the size of the patient population. Also, a significantly higher number of patients with ischemic cardiomyopathy were observed in the regional reference hospital. This selection bias was probably due to the coronary angiogram capabilities at the regional reference hospital which is absent in the other centers. Likewise, there were significantly more patients with atrial fibrillation in the regional reference hospital. However, persistent AF is not cited as a risk factor for developing an ACS in the ESC guidelines of 2023 [5]. The ECG data came from the doctors treating the patients, meaning there was a risk of interpretation bias, but this is limited by the ACS diagnosis being based on information from multiple sources including medical history, clinical examination, ECG, troponin, and coronary angiogram results. There was also a memorization bias due to patients being stressed and in pain when answering the MHS questions. Providing a negative response to the “pain not reproducible on palpation” question could also have been a source of selective reporting bias.

There were 28 false negatives, of which six had ST-segment elevation myocardial infarction, which confirms the need to perform an ECG within the first 10 min of ED admission regardless of where the patient is referred, ED-ICU or in the conventional sector.

Nevertheless, this study is strengthened by having involved several centers and a large sample size. This enabled an excellent NPV, in line with previous research. Furthermore, these results are reproducible, and the MHS can be easily performed by a nurse within a medical center.

This study reveals that the MHS is a useful tool for ED nurses to triage patients with chest pain and suspected ACS in an ED. This tool may prevent unnecessary transfer of non-ACS patients to the ED-ICU who could be appropriately managed in a conventional ED bed. These preliminary results need to be confirmed with an interventional study before considering their use in clinical practice.

## Figures and Tables

**Table 1:** Characteristics of the study centers.

	Tertiary referral hospital	Local Hospital 1	Local Hospital 2	Local Hospital 3
Number of visits over the inclusion period	56,566	49,000	19,691	29,740
Cardiologist on call 24 h a day 7 days a week	Yes	Yes	No	No
Onsite coronary angiography over the inclusion period	Yes	No	No	No

**Table 2:** Study population characteristics.

	Total population (*n* = 1045)	Center				*p* value
		Tertiary referral hospital (*n* = 460)	Local Hospital 1 (*n* = 319)	Local Hospital 2 (*n* = 92)	Local Hospital 3 (*n* = 174)	
Male *n* (%)	576 (55.1)	245 (53.3)	186 (58.3)	43 (46.7)	102 (58.6)	0.14
Female *n* (%)	469 (44.9)	215 (46.7)	133 (41.7)	49 (53.3)	72 (41.4)	
Age; mean ± standard deviation	53.00 ± 20.32	53.32 ± 20.32	53.64 ± 20.09	5.64 ± 20.09	51.21 ± 18.44	0.62
GP, physician, or self-referral to the emergency department; *n* (%)	754 (72.2)	256 (55.7)	278 (87.1)	72 (78.3)	149 (85.1)	< 0.001
Emergency call center referral to the emergency department; *n* (%)	291 (27.8)	204 (44.3)	41 (12.9)	20 (21.7)	26 (14.9)	
Typical chest pain; *n* (%)	890 (85.2)	392 (85.2)	271 (85.0)	73 (79.3)	154 (89.0)	0.21
Cardiovascular risk factor: age; *n* (%)	394 (37.7)	166 (36.1)	127 (39.8)	42 (45.7)	59 (33.9)	0.19
Cardiovascular risk factor: hereditary; *n* (%)	71 (6.8)	33 (7.2)	18 (5.6)	4 (4.3)	16 (9.1)	0.35
Hypertension; *n* (%)	286 (27.4)	113 (24.6)	84 (26.3)	28 (30.4)	61 (35.1)	0.05
Dyslipidemia; *n* (%)	182 (17.4)	65 (14.1)	65 (20.4)	15 (16.3)	37 (21.3)	0.06
Diabetes; *n* (%)	90 (8.6)	38 (8.3)	29 (9.1)	7 (7.6)	16 (9.2)	0.94
Obesity; *n* (%)	28 (2.7)	11 (2.4)	8 (2.5)	0 (0.0)	9 (5.2)	0.08
Smoking; *n* (%)	345 (33.0)	130 (28.3)	108 (33.9)	24 (26.1)	83 (47.7)	< 0.001
Ischemic cardiomyopathy; *n* (%)	155 (14.8)	92 (20.0)	31 (9.7)	11 (12.0)	21 (12.1)	< 0.001
Peripheral artery disease; *n* (%)	16 (1.5)	7 (1.5)	4 (1.3)	0 (0.0)	5 (2.9)	0.36
Atrial fibrillation; *n* (%)	62 (5.9)	39 (8.5)	12 (3.8)	4 (4.3)	7 (4.0)	0.02
Sleep apnea syndrome; *n* (%)	6 (0.6)	2 (0.4)	0 (0.0)	0 (0.0)	4 (2.3)	0.02

**Table 3:** Accuracy measures for the MHS as a triage tool with a cutoff of ≤ 2.

	Overall	Tertiary referral hospital	Local Hospital 1	Local Hospital 2	Local Hospital 3
Positive predictive value (%) [95% CI]	17.0 [13.3–20.6]	20.1 [14.4–25.8]	15.8 [8.7–23.0]	20.5 [8.5–32.4]	8.2 [1.9–14.5]
Negative predictive value (%) [95% CI]	95.6 [94.0–97.2]	95.9 [93.6–98.3]	93.6 [90.3–96.8]	100.0 [100.0–100.0]	97.0 [93.7–100.0]
Sensitivity (%) [95% CI]	71.1 [61.4–79.2]	77.6 [63.9–87.1]	53.3 [36.2–69.7]	100.0 [65.0–100.0]	66.7 [35.1–88.0]
Specificity (%) [95% CI]	64.3 [61.2–67.3]	63.3 [58.5–67.8]	70.6 [65.1–75.5]	57.8 [47.1–67.9]	59.4 [51.8–66.6]
Positive likelihood ratio	1.995 [1.712–2.325]	2.111 [1.733–2.570]	1.813 [1.241–2.650]	2.371 [1.843–3.051]	1.642 [0.998–2.700]
Negative likelihood ratio	0.499 [0.327–0.615]	0.335 [0.210–0.600]	0.661 [0.448–0.976]	0.000 [0.000–0.000]	0.571 [0.221–1.426]

*Note:* The MHS ≤ 2 rules out ACS for emergency department triage with a negative predictive value of 95.6% (94.0–97.2).

**Table 4:** Comparing mean Marburg Heart Score and acute coronary syndrome diagnosis between referral types.

	GP, physician, or self-referral (*n* = 754)	Emergency call center referral (*n* = 291)	*p* value
Mean MHS	2.09 ± 1.19	2.43 ± 1.25	< 0.001

Patients diagnosed without ACS	697 (92.4)	251 (86.3)	0.002
Patients diagnosed with ACS	57 (7.6)	40 (13.7)	

*Note:* The mean Marburg Heart Score (MHS) was significantly higher in patients referred to the ED by the emergency call center. A significantly higher number of patients referred to the ED by the emergency call center were diagnosed with acute coronary syndrome (ACS). *p* values less than 0.05 are statistically significant.

## Data Availability

The data that support the findings of this study are available from the corresponding author upon reasonable request.
